# Continent Ileostomy as an Alternative to End Ileostomy

**DOI:** 10.1155/2020/9740980

**Published:** 2020-01-10

**Authors:** Xian-rui Wu, Hao-xian Ke, Ravi P. Kiran, Bo Shen, Ping Lan

**Affiliations:** ^1^Department of Colorectal Surgery, The Sixth Affiliated Hospital, Sun Yat-Sen University, Guangzhou 510655, China; ^2^Guangdong Provincial Key Laboratory of Colorectal and Pelvic Floor Diseases, The Sixth Affiliated Hospital of Sun Yat-Sen University, Guangzhou, Guangdong 510655, China; ^3^Division of Colorectal Surgery, New York-Presbyterian Hospital, Columbia University Medical Center, New York 10032, USA; ^4^Department of Gastroenterology/Hepatology, The Cleveland Clinic Foundation, Cleveland, Ohio 44111, USA

## Abstract

Continent ileostomy (CI) was once a prevalent surgical technique for patients who required total proctocolectomy but then gave way to ileal pouch-anal anastomosis (IPAA) after 1980. Although IPAA has been the gold standard procedure preferred by most patients when total proctocolectomy is required, due to its imitation of physiological function of rectum and preserved function of anus, various complications have been observed with a relatively high rate of morbidity that could affect pouch longevity. Once serious complications such as pelvic abscesses and/or fistula occur, the pouch often needs to be removed. In addition, for some patients with a shortened small intestine or foreshortened mesentery, it is impossible for the ileal pouch to reach the pelvic floor, thus making the creation of an IPAA difficult. Previously, most of these patients would be referred for an end ileostomy, with an associated poor quality of life. In this circumstance, we propose that CI may deserve a reappraisal and serve as an alternative. In this article, we review the indications, contraindications, technique evolution, and outcomes of CI.

## 1. Introduction

The continent ileostomy (CI), first reported by Nils G. Kock in 1969, was an option for patients with ulcerative colitis (UC) or familial adenomatous polyposis (FAP) when they were referred to have a total proctocolectomy and permanent end ileostomy previously [1]. The CI technique involves a valve mechanism with advantages of the elimination of an external appliance and promotion of body image, therefore improving patients' quality of life (QOL) [2, 3]. Because of its tangible satisfaction among patients, CI was prevalent in the late 1960s and early 1970s. However, several problems were observed in the short-term or long-term period after the operation [4, 5]. Reported early complications included leakage from suture lines, necrosis of the intussuscepted valve, and hemorrhage from the various suture lines, while late complications included prolapse, fistulas, and particularly valve slippage [6]. Valve slippage was one of the main reasons for reoperation, which was often required in these patients. CI was later supplanted by restorative proctocolectomy and ileal pouch-anal anastomosis (IPAA), which has a lower need for revisions and is less technically challenging [7–9]. Comparison characteristics of CI and IPAA were summarized in Table 1. Currently, CI is used in patients who are not suitable for IPAA or have an unsatisfactory function of the IPAA, which mainly results from perianal or anal disease, poor sphincter strength, or pouch vaginal fistulas [10]. As new techniques have developed, CI is now associated with fewer complications and lower revision rates than previously [11]. In this article, we have elaborated on the indications, contraindications, technique evolution, and outcomes of the CI procedure.

## 2. Indications for Continent Ileostomy

CI can be performed either as a primary procedure for patients requiring restorative proctocolectomy or as a salvage procedure for those who have failed pelvic pouches.

For patients who have not previously undergone abdominal surgery, the construction of a CI is mainly recommended to patients with difficult anatomy, such as a short small bowel mesentery, dysfunctional sphincter, or irradiated pelvis. A short small bowel mesentery, which makes the small bowel difficult to reach the pelvic floor, precludes an IPAA. Patients with dysfunctional anal sphincter are also not suitable for an IPAA since this is associated with poor postoperative functional outcomes. In addition, an irradiated pelvic floor, inducing inflammation or fibrosis, prevents access to the pelvis and hence the construction of an IPAA [12]. Selected patients with CD with large bowel involvement and a normal small bowel can occasionally be considered for CI after a full discussion of the implications. UC patients with complicated perianal fistulas which hamper the construction of an IPAA may also be candidates for CI.

On the other hand, CI can be considered an option for patients when IPAA is contraindicated, or in IPAA failure when reduced small intestinal length impossible to reach pelvic floor prevents redoing the pelvic pouch surgery. IPAA is currently the gold standard for patients following a total proctocolectomy and is associated with fewer restrictions in sports and sexual activities than CI, offering a better QOL [13]. However, Mukewar et al. [14] reported that J-pouch and S-pouch showed no significant difference in the rate of pouch-associated hospitalization and pouch failure from CI. Wasmuth and Myrvold [15] also demonstrated that the long-term failure rate of IPAA was 11.4% at 20 years, similar to that of CI, 11.6% (Table 1). Considering the ability to control feces and gas, when patients are not suitable for IPAA or have a failed IPAA, CI could be considered an alternative to an end ileostomy.

When pouch failure occurs and a redo IPAA is not feasible or desirable, conversion of the original pouch to CI is preferred when expertise is available, since this allows conservation of intestinal length [16]. While conversion of IPAA to CI is complicated and challenging, studies evaluating the feasibility of converting an IPAA to CI reported good outcomes [17, 18]. A retrospective study found no substantial difference in revisions, diurnal, and nocturnal frequencies of intubation for patients who had CI after failed IPAA and those who had CI without having a previous restorative procedure [19]. Lian and colleagues [20] however reported that the long-term complication rate was high for patients with CI after a failed IPAA and 45% of the patients needed revision surgery, although the QOL was good overall after conversion. Pelvic sepsis, previous surgery, adhesion formation, and pelvic fibrosis could also make dissection impossible or too risky for conversion. Thus, careful consideration should be given to patients evaluated for the conversion of failed IPAA to CI since a second operation impacts potential morbidity and further risk of failure [20, 21]. Patients who choose CI should be fully informed of the possibility of further surgical revisions and the risk of short bowel syndrome.

Patients with problems of an end ileostomy and those seeking to avoid an external appliance and hence an improved body image can also be considered for CI.

Indications for a CI was elucidated in Table 2.

## 3. Contraindications for Continent Ileostomy

Whether CD patients should be offered a CI remained controversial [22]. Previous studies often included some CD patients primarily because of undetermined diagnosis before surgery and reported that postoperative outcomes of CI in these patients were poor [2, 23]. These patients had high rates of fistula and resistant or recurrent pouchitis leading to pouch failure. Aytac et al. [23] evaluated outcomes for CD patients with CI as intentional, defined as a diagnosis of CD before CI; and delayed, defined as diagnosis after CI. They found that outcomes of CI in patients with CD were poor, with 48% pouch survival at 20 years, regardless of the timing of CD diagnosis. However, another study from the same institution reported that valve slippage and revision rates in CD patients who received a CI were not different from those with UC [2].

Patients potential for short small bowel syndrome are not recommended for a CI since a relatively long segment of small bowel is required for constructing the pouch, which will increase the risk for short bowel syndrome if the operation were to fail.

For acute severe colitis, CI is avoided in the acute setting given the additional time required in these often sick patients. Subtotal colectomy with an end ileostomy is the initial choice with a CI considered at the second stage of the surgery after the patients recover from their illness.

It is important to note that the surgical procedure for CI is complex and the ability to handle intubation is acquired after surgery. Patients should be well informed prior to surgery, and the procedure should be contraindicated to those who are unable to understand such essential information. In addition, children and patients with learning difficulties or those with their extremities precluding intubation are unsuitable for CI.

Contraindications for a CI was elucidated in Table 2.

## 4. Construction of a KOCK Pouch

**Figure 1:**
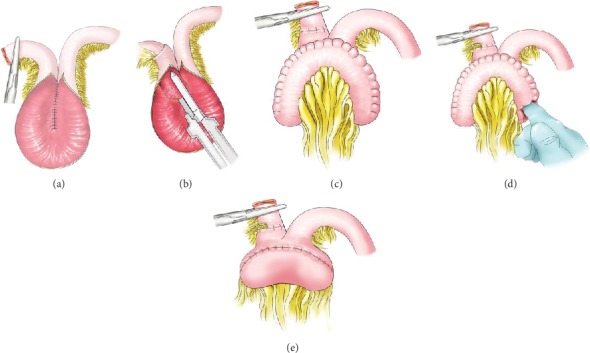
Construction of a Kock pouch reservoir.

The conventional continent ileostomy described by Kock is referred to as a Kock pouch in this review. In brief, after proctocolectomy, the penultimate 30-45 cm of the ileum is used to create a J- or S-shaped reservoir, and the terminal 15 cm used to create a nipple valve and exit conduit that traverses the abdominal wall. In obese patients, a greater length of small bowel should be reserved for the exit conduit. In the J-pouch design, the antimesenteric border of the two proximal 15 cm segments is apposed with suture after which, the lumen is opened. The posterior layer is sutured (Figure 1(a)). After removing peritoneum and fat from the mesentery, the distal 10 cm segment is invaginated to form an artificial (nipple) valve (Figure 1(b)). The nipple is then fixed in place and then to the inside wall of the pouch using staples and strengthened by sutures to hold the valve in place. The intestinal wall is then folded and sutured to close the pouch (Figure 1(c)). It is important that the both ends of banana-shaped pouch must be pushed by a finger to go through the mesentery (Figure 1(d)); hence, a spherical pouch will be created (Figure 1(e)). The end of the intestine is brought out through an aperture on the abdominal wall, and a catheter placed in the reservoir for draining temporarily [24].

## 5. Construction of a Barnett Continent Intestinal Reservoir

**Figure 2:**
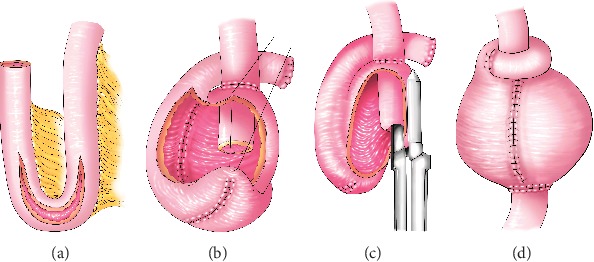
Construction of a Barnett continent intestinal reservoir.

To deal with the high rate of valve slippage and fistula formation, Barnett created an isoperistaltic valve and collar [25] Approximately 60 cm of ileum is used to construct the Barnett continent intestinal reservoir (BCIR), which consists of a 10 cm segment of the intestine as a living collar around the external circumference of the valve, 30 cm for pouch body, 12 cm for an isoperistaltic valve, and 5 cm for the conduit through the abdominal wall. The construction of the pouch body is similar to the Kock pouch (Figure 2(a)), while the efferent limb (12 cm segment) is invaginated to make an isoperistaltic valve 5 cm in length, held in place by staples and sutures (Figures 2(b) and 2(c)). A 10 cm distal segment is then anastomosed to the external circumference of the valve to form a living collar, which reportedly enhances valve stability and prevents slippage (Figure 2(d)). The proximal edge of the small bowel is divided, and the ileum and pouch are connected by an end-to-side anastomosis. The terminal end of the intestine is brought out through the abdominal wall for access to the pouch.

## 6. Construction of a T-Pouch

**Figure 3:**
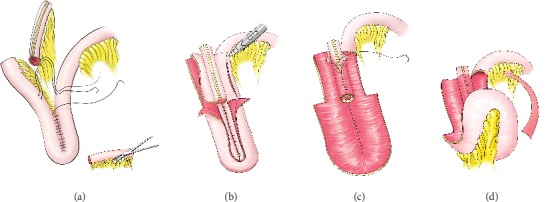
Construction of a T-pouch.

A total of 55 cm of ileum is needed for the T-pouch. The distal 15 cm segment of small bowel makes an efferent antireflux valve and ostomy, while the proximal 40 cm forms the pouch body, creating a “U” shape with each 20 cm limb. The blood supply to the mesentery of the distal 15 cm of small intestine is preserved with avascular mesenteric windows opened to increase the mobility of the bowel and to create space for sutures. The intestine is then used to make a valve segment between the two limbs of the U. The mesenteric border of the two limbs is approximated while the valve segment is fixed by a series of interrupted sutures passing through the avascular mesenteric windows (Figure 3(a)). The two limbs of the U-shaped intestine are then opened from the bottom adjoining the mesentery to the internal valve opening with the incision crossing to the antimesenteric border (Figure 3(b)). The internal edges of the flaps are approximated, and the upper interior part of the flaps encompassed into the interpolated valve segment (Figure 3(c)). Finally, the pouch is closed by folding the bottom upwards and sutured (Figure 3(d)). The terminal portion is then brought out through the abdominal wall as a stoma.

## 7. Early Postoperative Complications

Surgical complications occurred frequently in the initial reports of CI but later decreased due to refined techniques and increasing experience. Short-term complications include hemorrhage from the suture lines, anastomotic leakage, ischemia or necrosis of the intussuscepted valve, and intestinal obstruction of the small bowel with adherence to the pouch and fistula formation, while long-term complications include valve slippage and prolapse, fistula, volvulus, perforation hernia, valve stenosis, and pouchitis [4].

Early complications can be severe and even lead to pouch excision. However, most patients are able to convalesce and keep their pouch. Parc et al. [5] reported that 35% of patients with CI suffered from early complications, including urological problems, abdominal wall or intraabdominal abscesses, enterocutaneous fistula, necrosis of stoma, partial slippage of the valve, peritoneal hemorrhage, and peritonitis. Most of the early complications could be managed, none required excision of the CI, and there was no mortality in this study. Early morbidity was similar to that of IPAA [26, 27].

## 8. Long-Term Adverse Sequelae of Continent Ileostomies

Similar to our proposal for ileal pouch disorders, the long-term adverse sequelae of CI could also be classified as structural, inflammatory, functional, and neoplastic complications [28]. (Table 3).

### 8.1. Structural Complications

#### 8.1.1. Valve Malfunction

Valve malfunction includes slippage, prolapse, stenosis, and necrosis of the valve, which can cause difficulty in intubation, incontinent stoma, or even pouch failure. The weakest point of the valve is at the mesenteric aspect where intussusception produces a large bulk of fatty mesentery that prevents the two walls of the valve from firmly attaching to each other [29, 30].

Nipple-valve slippage (29.7%) has been recognized as one of the most frequent causes for pouch revision or failure in patients with Kock pouch, followed by fistula formation (25.2%) [2, 6, 31]. It often occurs within the first year of pouch construction, with 43.9% reported in Kock pouch and 62.5% in BCIR [2, 32]. Slippage is impossible in T-pouch because of its unique construction of efferent antireflux valve. However, malfunction of the valve could also lead to incontinence if a relatively short segment of bowel is used for construction of the valve, while difficult intubation occurs if the valve is long. Valve slippage is less frequent in patients with BCIR than Kock pouch, 6.3%, compared to 29.7% in Kock pouch [2, 32]. However, a study from the Cleveland Clinic-enrolled patients referred to Kock pouch or BCIR demonstrated that the slippage rate for this subgroup of patients with isoperistaltic valve (23.7%) was similar to those of the anisoperistaltic group (25%) [2].

Valve prolapse occurs as complete slippage of the nipple valve when there is no adherence to the abdominal wall, often due to the fascial defect which is made too large at surgery. Difficulty with intubation often occurs, and surgical repair is usually necessary. Necrosis of the valve occurs from ischemia, often due to excessive sutures for valve fixation.

With modifications in surgical technique, postoperative complication rates of the nipple valve have been reported to decrease from 41.1% to 4.8% as reported by Ecker [33]. The rates of revision for nipple valve malfunction vary depending on the surgical approach. Early and later studies differ with regard to the rate of revisions of patients with Kock pouch due to nipple valve malfunction, 54% during the period from 1967 to 1974 versus less than 10% during the period from 1975 to 1984 [34]. Kaiser [35] reported a 15% need for valve revision of T-pouch.

#### 8.1.2. Fistula

Fistula can develop anytime after surgery and occur in the valve, pouch, afferent limb, or in the collar in patients with BCIR [4, 32]. When fistula develops at the base of the valve, the intestinal contents can bypass the valve, and hence, continence is affected. Fistula may result from technical issues during valve construction. One recognized cause for fistula formation is the use of nonabsorbable mesh, previously introduced to strengthen the pouch [36]. In one study, the frequency of fistulas has been reported to be as high as 25.2% for patients with mesh, compared to 14% without mesh [2]. CD is another risk factor for fistula formation, which is also associated with a higher rate of pouch excision. Refractory fistula especially when it presents in the pouch or efferent limb indicates the possibility of CD. The rate of fistula formation varies with the surgical procedure, 25.2% in Kock pouch and 10.2% in BCIR [2, 32]. In most circumstances, fistula can be managed with surgical revision; however, pouch excision might be inevitable when revisions fail. It was reported that fistula was one of the major reasons for pouch excision [2].

#### 8.1.3. Stoma-Related Issues

Approximately 10% of patients with Kock pouch develop stoma stricture, compared to 25% of patients with a T-pouch [2, 35]. Parastomal hernia occurred in 15.5% of patients with Kock pouch, and 1.5% of patients with BCIR [2, 32]. Difficulty in intubation is the most frequent complaint of these patients. Surgery might be required if conservative methods fail [37]. To prevent stoma stricture and enhance the continence of stoma, it was reported that transcutaneous implant evacuation system, TIES device, might be applied [38]. It is a ring-like titanium implant with an upper solid part placed outside the abdominal skin for an attached lid and with a lower part placed in the subcutis, while the saddle-like part is a mesh, which is designed to promote the healing of the skin and intestine. The application of such a device remains controversial [39], and further studies are warranted.

### 8.2. Inflammatory Complications

#### 8.2.1. Pouchitis

Pouchitis, one of the major nonoperative complications, is the inflammation of pouch mucosa, usually accompanied by overgrowth of bacteria. The etiology of pouchitis has not been clearly elucidated. Several hypotheses for the development of pouchitis have been proposed, such as recurrence of UC, dysbiosis, deprivation of short-chain fatty acids, mucosal ischemia, host gene susceptibility, and immune dysregulation [40, 41]. The clinical presentations include a thick effluent, excess excretion, malodor, and bleeding. Patients may have abdominal pain, distention, diarrhea, and fever. The frequency of pouchitis in patients with Kock pouch varies from 26.4% to 29% [2, 42]. Notably, CD patients are more prone to resistant or recurrent pouchitis at a rate of 47.6%, likely requiring pouch removal, which has been shown to be as high as 26% [2, 43]. Antibiotics, probiotics, and continuous catheter drainage may be beneficial. Infliximab and ustekinumab have also been shown to be effective for pouchitis in some cases [44–46].

#### 8.2.2. Crohn's Disease of the Pouch

CD of the pouch can occur *de novo* after the construction of CI or in some cases, the pouch can intentionally be created in some CD patients without the involvement of small bowel or perianal disease. Clinically, CD of the pouch can be classified into inflammatory, fibrostenotic, or fistulizing phenotypes. As mentioned, CD was one of the main risk factors of pouch failure, and patients were 4.5 times more likely to develop pouch failure compared to those with FAP or UC in one study [2]. The diagnosis of CD of the pouch can be based on findings noted in the previous colectomy specimen and particular characteristics such as transmural ulceration of pouch, inlet stricture, afferent limb ulcers, and fistula formation. CD manifestations should be distinguished from other conditions such as nonsteroidal anti-inflammatory drug-induced ileitis and backwash ileitis. Due to the rarity of CD of the pouch in CI patients, the experience with diagnosis and management of CD in IPAA could be applied to CI patients [28].

### 8.3. Functional Complications

#### 8.3.1. Short Bowel Syndrome

Since a part of the intestine has to be removed in patients with failed IPAA, and approximately 60 cm of intestine is needed for the construction of CI, patients being considered for CI may face with the risk of short bowel syndrome and its associated complications such as diarrhea, dehydration, weight loss, and nutrition deficiency [47]. Patients with short bowel syndrome are vulnerable to kidney failure and septic shock, even leading to death [23]. The length of residual small intestine alone was insufficient to accurately determine the degree of dysfunction of the bowel. Therefore, surgeons should take this severe complication into consideration. Available surgical therapies are limited for short bowel syndrome and are aimed at reducing motility, lengthening the native small bowel, or small bowel transplantation [48].

### 8.4. Dysplastic and Neoplastic

IBD and FAP are associated with an increased risk of dysplasia and cancer [49]. While proctocolectomy decreases this risk, neoplasia can still develop and occur in the pouch [2, 28]. A few cases of cancer in the pouch have been described, the etiology of cancer has however not been elaborated due to the paucity of data about the natural history of dysplasia and effective surveillance in patients with a pouch. Generally, endoscopic surveillance is recommended for patients with FAP or IBD even after proctocolectomy [50]. However, whether endoscopic surveillance is necessary or adequate remains debatable.

## 9. Pouch Revision and Pouch Survival

### 9.1. Pouch Revision

Several retrospective studies on CI with a large sample size reported that the rate of pouch revision was high, mainly due to valve dysfunction, mesh usage, fistula formation, and/or anastomotic leakage [2, 34]. Kock et al. [34] reported 97% of 273 patients required revision during the study period from 1975 to 1984. A multicenter study demonstrated 20.9% of patients with BCIR needed major or minor revision after a mean follow-up of 2.2 years.(range, 0.8-4.8 years) [32]. Kaiser [35] indicated that 30% of patients with T-pouch eventually required revision at a median follow-up of 6.2 years (range, 0.8-11 years). In general, revision rates in patients with CI have been variably reported as 21% to 70% after 1984 [32, 34, 35, 51] (Table 1). However, the majority of patients did well after revision, and a third operation was infrequently needed. Several confounding factors make comparisons of revision rates between the 3 CI techniques difficult [52]. Interestingly, when it comes to the comparison of pouch revision rate between CI and IPAA, it was reported that revision rate of CI was not inferior to that of IPAA [9, 53, 54]. Wasmuth and Myrvold [15] reported a rate of 38% in CI versus 31% in IPAA (Table 1).

### 9.2. Pouch Survival

Most patients with CI can maintain a functional pouch for a prolonged period. Nessar et al. [2] reported only 16.6% of patients with Kock pouch lost their pouch after a median follow-up of 11 years. At 10 years after surgery, 87% of all patients maintained their pouch, while 77% maintained the pouch at 20 years. For BCIR, data regarding the long-term follow-up results are still lacking. Mullen et al. [32] reported 92.2% of 510 patients who underwent BCIR in four centers between 1988 and 1991 had fully functioning pouches at least one year after initial surgery. For T-pouch, Kaiser [35] reported 10% of patients had their pouch excised during the first 10 years. However, most patients with T-pouch were converted from a Kock pouch or J-pouch, while primary T-pouch creation was rare. For IPAA, 8.5% of patients with IPAA have pouch failure on follow-up of more than 5 years, with up to 10% having failure at 10 years, leading to end ileostomy according to one study [55, 56]. With continued follow-up, the pouch failure rate could reach 15% or more at 20 years [9]. When comparing pouch survival rate between CI and IPAA (Table 1), Wasmuth and Myrvold [15] showed that the durability of both was similar.

Several clinicopathological factors influencing pouch survival have been reported. A multivariate model to assess factors about pouch excision in patients with CI showed that the underlying disease, gender, fistula development, and body mass index (BMI) were potential risk factors [2]. Patients with CD or indeterminate colitis had a higher risk of pouch failure than patients with UC or FAP. In addition, female patients were more likely to develop pouch failure. A study of patients with BCIR demonstrated that valve slippage and pouch or valve fistulas seemed to be the major reasons for pouch failure [32].

## 10. Functional Outcomes and Quality of Life

Previous studies showed that the majority of patients who responded to questionnaires declared satisfaction to the outcomes of surgeries, regardless of the types of CI (Kock pouch, BCIR, or T-pouch) [2, 32, 35]. The function of pouch could be evaluated by ease of intubation and control of gas/stool. For all designs of CI, intubation was easy or encumbered when problems occurred in the stoma or pouch, such as stricture of stoma, valve slippage, and prolapse. Most patients with CI were continent with slight seepage in some individuals. Compared with end ileostomy, patients with Kock pouch had a lower frequency of pouch emptying both diurnally and nocturnally and less abdominal pain [2]. For most patients with BCIR, the frequency of intubation was moderate in the daytime and rare at night, and the function of the continent stoma was acceptable [32]. When Kock pouch or J-pouch failed, conversion to T-pouch might also ameliorate gross incontinence, leakage of stool, and mucus seepage [35].

For QOL, a study suggested that patients with Kock pouch were less likely to report dietary, social, work, and sexual restrictions than end ileostomy patients who needed more antidiarrheal medication and fiber intake and that hospitalization and pouch complications occurred less frequently in patients with Kock pouch [2]. With BCIR, Mullen et al. [32] indicated that most patients reported a better QOL after surgery. Kaiser [35] reported that for T-pouch after 10 years, follow-up patients were content after surgery, with less work, social, dietary, and sexual restrictions.

## 11. Summary and Recommendation

**Figure 4:**
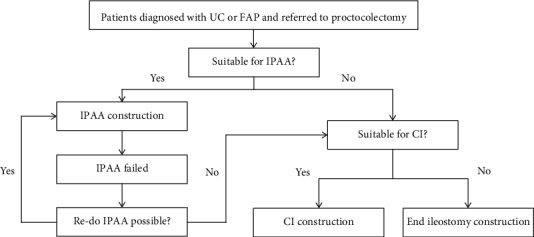
Proposed algorithm for selection of a proper reservoir in patients who require a total proctocolectomy.

Compared with end ileostomy, CI has noticeable advantages in QOL. The major merit of CI over end ileostomy is its ability to make patients free of external appliance and thus improve body image. For patients with a failed IPAA, when construction of a new IPAA is not feasible or desirable, CI may be an option (Figure 4). Although the operation is technically challenging and may be associated with short-term and long-term complications, the majority of patients are happy with the results of the procedure and are able to retain their pouch for a long time [57]. Surveys based on questionnaires demonstrate that patients with CI are satisfied and would like to recommend CI to other patients when compared to those with an end ileostomy [2, 58, 59].

In conclusion, CI is associated with acceptable outcomes in experienced hands and should be considered an alternative to end ileostomy and IPAA. CI is an option for patients with a failed IPAA when a new IPAA is not feasible or desirable.

## Figures and Tables

**Table 1:** General considerations of continent ileostomy and ileal pouch-anal anastomosis.

	Continent ileostomy	Ileal pouch-anal anastomosis
Surgical configuration	Construction of a pouch and valve, and creation of a continent stoma	Construction of a pouch and anastomosis to the anus
Mortality	Rare	Rare
Pouch failure rate	5-20% [2, 15, 32, 35, 51]	6-16% [9, 10, 56, 60]
Pouch revision rate	21-70% [32, 34, 35, 51]	5-89% [9, 53, 54]
Quality of life	Mostly satisfied	Mostly satisfied

**Table 2:** Indications and contraindications for a continent ileostomy.

Indications
Unsuitable anatomy for IPAA
Short small bowel or mesentery unable to reach pelvic floor
Sphincter excision or malfunction
Pelvic radiation
Selected Crohn' s disease
Perianal fistulas
Failed IPAA
Functioning or dysfunctional conventional ileostomy
Contraindications
Most patients with Crohn's disease
Desmoid disease
Potential risk of short bowel syndrome
Exigent surgery for acute severe colitis
Inability to manage stomal intubation

IPAA: ileal pouch-anal anastomosis.

**Table 3:** Classification of long-term complications of continent ileostomy.

Structural	Valve malfunction, such as valve slippage, prolapse, and stenosis
	Pouch fistula
	Stoma-related problems, such as stomal stenosis and parastomal hernia
Inflammatory and infectious	Pouchitis
	Crohn's disease of the pouch
Functional	Short bowel syndrome
Dysplastic and neoplastic	Dysplasia or cancer of the pouch

## Abbreviations

BCIR: Barnett continent intestinal reservoir
BMI: Body mass index
CD: Crohn's disease
CI: Continent ileostomy
FAP: Familial adenomatous polyposis
IBD: Inflammatory bowel disease
IPAA: Ileal pouch-anal anastomosis
QOL: Quality of life
UC: Ulcerative colitis.
